# New Cu-Free Ti-Based Composites with Residual Amorphous Matrix

**DOI:** 10.3390/ma9050331

**Published:** 2016-04-30

**Authors:** Mircea Nicoara, Cosmin Locovei, Viorel Aurel Șerban, R. Parthiban, Mariana Calin, Mihai Stoica

**Affiliations:** 1 Materials and Manufacturing Engineering Department, Politehnica University Timisoara, P-ta Victoriei 2, Timisoara RO-300006, Romania; mnicoara@gmail.com (M.N.); cosmin.locovei@gmail.com (C.L.); viorel.serban@upt.ro (V.A.Ș.); 2 Institute for Complex Materials, IFW Dresden, Helmholtzstr. 20, Dresden D-01069, Germany; p.ramasamy@ifw-dresden.de (R.P.); m.calin@ifw-dresden.de (M.C.)

**Keywords:** Ti-based composites, phase separation, Cu-free alloy

## Abstract

Titanium-based bulk metallic glasses (BMGs) are considered to have potential for biomedical applications because they combine favorable mechanical properties and good biocompatibility. Copper represents the most common alloying element, which provides high amorphization capacity, but reports emphasizing cytotoxic effects of this element have risen concerns about possible effects on human health. A new copper-free alloy with atomic composition Ti_42_Zr_10_Pd_14_Ag_26_Sn_8_, in which Cu is completely replaced by Ag, was formulated based on Morinaga’s d-electron alloy design theory. Following this theory, the actual amount of alloying elements, which defines the values of covalent bond strength Bo and d-orbital energy Md, situates the newly designed alloy inside the BMG domain. By mean of centrifugal casting, cylindrical rods with diameters between 2 and 5 mm were fabricated from this new alloy. Differential scanning calorimetry (DSC) and X-rays diffraction (XRD), as well as microstructural analyses using optical and scanning electron microscopy (OM/SEM) revealed an interesting structure characterized by liquid phase-separated formation of crystalline Ag, as well as metastable intermetallic phases embedded in residual amorphous phases.

## 1. Introduction

Titanium alloys still raise the highest research interest among biomaterials for orthopedic and dental applications since they possess the most favorable combination of properties [1,2]. In terms of mechanical properties, titanium alloys have good mechanical strength and ductility [3], as well as acceptable wear resistance [4]. They also have excellent resistance to bio-corrosion [4], they stimulate the proliferation of new cells and tissues [1], and reported incidence of adverse toxic, irritating, inflammatory, or allergic reactions produced by released elements is relatively moderate [1]. Therefore, over the years, some titanium alloys like Ti-6Al-4V and Ti-6Al-7Nb became standard solution for medical implants [1]. In spite of undeniable advantages, classical crystalline alloys based on titanium still present some inadequate properties: certain alloying elements such as vanadium, aluminum, nickel, *etc*. could release harmful metallic ions inside human body, having well established allergenic or cytotoxic effects [5,6]. They also have considerable higher rigidity than cortical bone, which is responsible for stress shielding effects of orthopedic implants and degradation of mineral density in patients’ bones (osteopenia) and still present relatively low bioactivity in relation with human tissues [1,3,4,6,7].

Development of new amorphous or composite amorphous-crystalline alloys fabricated by means of ultra-rapid melt cooling represents a most promising improvement of properties [8,9,10]. Amorphous alloys, known also as metallic glasses, have improved corrosion resistance, better mechanical properties such as tensile strength and wear resistance, and, even more importantly for medical implants, they have a lower Young’s modulus than crystalline alloys [6,11]. It may be considered that the lack of grain boundaries, segregations, and other structural heterogeneities, are responsible for these considerable enhancements with large potential for applications [8,9].

Metallic glasses could offer also technological advantages based on their good formability. Plastic deformations could be performed at high ratio at temperature levels between glass transition (Tg) and crystallization (Tx), similar to thermoplastic forming [8]. This special feature has been valorized for massive amorphous alloys with thickness over 1 mm, called bulk metallic glasses (BMGs), allowing fabrication of components with complex shapes by means of different deformation methods [8,9,12]. Very recently, it was shown that bulk porous Ti-based BMGs, replicating the topological features of the cortical human bones and with mechanical properties close to them, can be successfully prepared by thermoplastic forming of amorphous alloy powders [13].

The metallic glasses were discovered only in the last half century [14]. Among them, the titanium-based amorphous alloys are newcomers [15]. Reports of titanium—zirconium glasses with additions of different alloying elements, especially nickel and beryllium, goes back until 1993 [16]. Upon increasing the glass forming ability (GFA) of newly designed alloys, fabrication of plates and rods with increased thickness or diameters is now possible [17]. The 1 mm conventional limit set for BMGs was achieved in 1998 with compositions based on Ti-Ni-Cu family, with additions of Zr, Be, or Sn [18,19]. More recent developments in design of amorphous alloys lead eventually to exceptionally massive alloys, with critical size up to 14 mm for the Ti_40_Zr_25_Cu_12_Ni_3_Be_20_ alloy [20] or even 32 mm for (Ti_41_Zr_25_Be_28_Fe_6_)_91_Cu_9_ [21]. However, application of these alloys as biocompatible materials remained problematic because serious concerns were raised by the presence of some elements that are detrimental to human health such as Ni, Be, Al, and Cu [6]. Therefore, recent developments are focused on formulations that are free of these harmful elements or at least reduced in proportion.

Elimination of nickel and beryllium was achieved in 2007 with the Ti-Zr-Cu-Pd family of compositions, based on a (Ti-Zr)_50_(Cu-Pd)_50_ concept and subsequent small additions of elements like Sn, Si, Ta, Nb, Co, or In [17,22,23,24,25,26,27,28]. This new approach is an important accomplishment allowing fabrication of components with critical dimensions up to 10 mm, simultaneously with elimination of nickel, which is one of the most allergenic elements, as well as beryllium, with its high cytotoxic effects.

Newly developed BMGs in the Ti-Zr-Cu-Pd system represent an important progress regarding possible biomedical applications, since they also have high corrosion resistance, mechanical strength up to 2000 MPa, and a Young’s modulus as low as 80 GPa [26,29,30]. Although biocompatibility seems to be improved in comparison with classic amorphous alloys Ti-6Al-V and Ti-45Ni [31], the most serious problem consists in the presence of copper, which is considered highly cytotoxic and could not be completely replaced so far as an amorphization element with other biocompatible additions.

**Figure A1:**
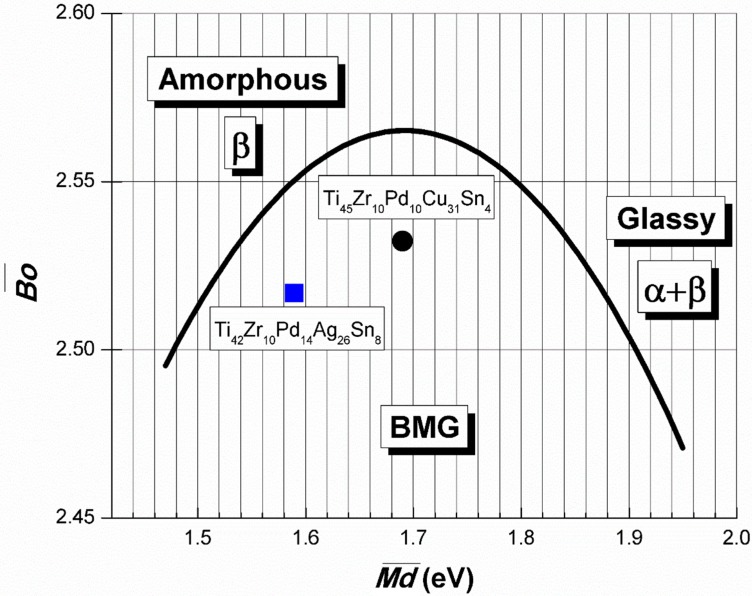
Positions of Ti_45_Zr_10_Pd_10_Cu_31_Sn_4_ and Ti_42_Zr_10_Pd_14_Ag_26_Sn_8_ alloys on the distribution chart of amorphous and glassy alloys, based on *B_o_* and *M_d_* parameters (adapted from reference [33]).

The main objective of present work was the development of new titanium-based amorphous alloys completely free of copper, starting from the well-known Ti_45_Zr_10_Pd_10_Cu_31_Sn_4_ BMG, which allow casting of amorphous rods with diameters up to 4 mm [26]. The designing of new copper-free alloys is based on the DV-Xα molecular orbital method [32,33]. The detailed concept of the alloy design is presented in Appendix A. There, the chart portion belonging to the area of bulk amorphous glasses is shown in Figure A1 and the list of $\bar{B_{o}}$ and $\bar{M_{d}}$ for some alloying elements are given in Table A1. The copper was replaced with silver, which could be considered more biocompatible. The optimization results are presented in Table A2. Regarding the biosafety, it has been long known that silver has an antimicrobial effect [34]. The biological effect of both copper and silver and the differences between them, showing that it is safer to use silver instead of copper, are reviewed in Appendix B. The amount of the other elements was also adjusted in order to keep the new composition within the amorphous bell (details in Figure A1, Appendix A). The resulted alloy has the composition Ti_42_Zr_10_Pd_14_Ag_26_Sn_8_ at %. Despite the fact that the newly designed alloy has a composition inside the BMG domain, the rapidly quenched samples show a complex microcrystalline structure, with only residual amorphous matrix.

## 2. Results

### 2.1. Thermodynamic Considerations Regarding the Master Alloy

**Figure 1:**
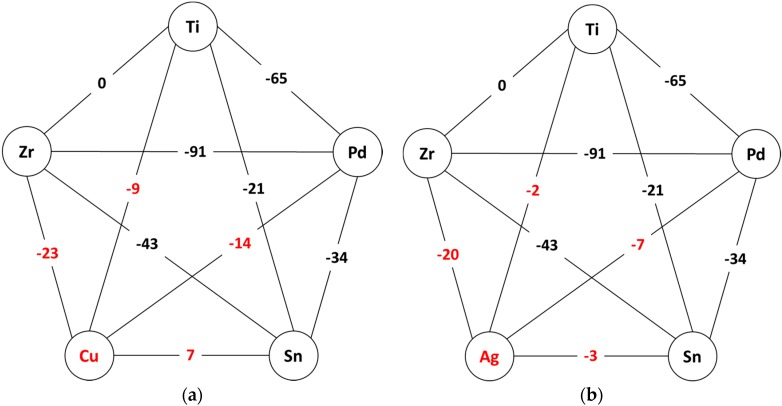
Mixing enthalpies *ΔH^mix^* between the atomic pairs of the alloy constituents (in kJ/mol), emphasizing (**a**) Cu-containing alloy; and (**b**) Ag-containing alloy.

Figure 1 shows schematically the enthalpy of mixing *ΔH^mix^* for each atomic pair of all alloy constituents, emphasizing the values characteristic to Cu-containing pairs (Figure 1a) and Ag-containing pairs (Figure 1b). The values were taken from reference [35], *i.e.*, calculated using Miedema’s model for the corresponding binary liquids.

As it can be observed, the mixing enthalpies are in general negative, with two exceptions: Ti-Zr, which is zero and Cu-Sn, which is +7 kJ/mol. The negative mixing enthalpies may indicate the possibility to form an alloy with a higher degree of dense randomly packed atomic configurations and multiple interatomic interactions in the liquid state, therefore being prone to amorphization. In fact, the starting Cu-containing composition Ti_45_Zr_10_Pd_10_Cu_31_Sn_4_, respects the three empirical rules as formulated by Inoue [15] and hence shows a good GFA. According to them, the replacement of Cu by Ag should be beneficial for glass-formation, because Ag has only negative heats of mixing with all elements. Experimentally observed, the new Ti_42_Zr_10_Pd_14_Ag_26_Sn_8_ master alloy is off eutectic and shows a large temperature interval over which liquid and solid coexists. The reasons will be analyzed later. Therefore the casting was performed with the alloy in the homogeneous liquid state, as it is described in Section 3. Even so, the resulted rods exhibit a clear phase separation. As it will be shown in the next section, one of the separated phases contains Ag up to 80 at %. Thus it is interesting to study in details the phase diagrams of binary alloys with Ag. Nevertheless, the analysis of only binary atomic pairs and binary alloys may not reflect accurately the situation in the quinary alloy, but in the absence of more elaborate models it may give at least a qualitative explanations of the observed behavior.

Ag is miscible with Pd over the entire compositional interval, in both liquid and solid state [36]. Therefore, the mixing with Pd should not put any problem. With Sn, Ag is miscible in the liquid state [36]. In the solid state, Sn is soluble in Ag over several at%. It forms also intermetalics like Ag_0.8_Sn_0.2_ and Ag_3_Sn. For more Sn the binary alloy decomposes and at ~96.5% Sn there it is a eutectic. The solubility of Ag in Sn below its melting temperature is almost zero. However, the total Sn content in the alloy is 8 at %, therefore only the Ag-rich part of the phase diagram might be of interest, and in this interval Sn may completely dissolve in Ag.

More problematic are the binary Ag-Zr and Ag-Ti alloys [36]. In both cases, the elements are miscible in the liquid state. In the solid state, Ag stabilizes the high-temperature phases of Ti and Zr. At room temperature, the solubility of Ag in Ti or in Zr, as well as Ti or Zr in Ag, is almost inexistent. Both Ag-Ti and Ag-Zr are not miscible in solid state over the entire compositional interval. However, there are few intermetallics, like AgTi and AgTi_2_ or AgZr and AgZr_2_. The AgTi compound is stable for a compositional interval of 50% ± 2%. As a detail, the Cu-Ti and Cu-Zr phase diagrams are characterized by the formation of far more intermetallic compounds—so in terms of competing crystalline phases and following the Inoue’s findings [15], it seems that the Cu-containing alloy may have a better GFA. Moreover, silver has a higher radius as compared with copper, *i.e.*, 0.144 nm as opposed to 0.128 nm, which makes the atomic radius mismatch between silver and titanium smaller, titanium having its metallic radius of 0.147 nm.

Ti and Zr are completely miscible as well in both liquid and solid states [36]. There is a large temperature interval (compositional dependent, with the minimum low temperature 600 °C and minimum high temperature 1554 °C), in which the ZrTi solid solution is of the type bcc β-Ti and below of the type hcp α-Ti. Pd and Ti form solid solution from room temperature up to the melting point (*i.e.*, up to at least 1400 °C, function of composition) in the Pd-rich side (Ti up to 20 at %), while above 50 at % Ti the bcc β-Ti type solid solution is stable over a large temperature interval (depending on the composition), showing an eutectoid decomposition for 90% Ti [36].

**Figure 2:**
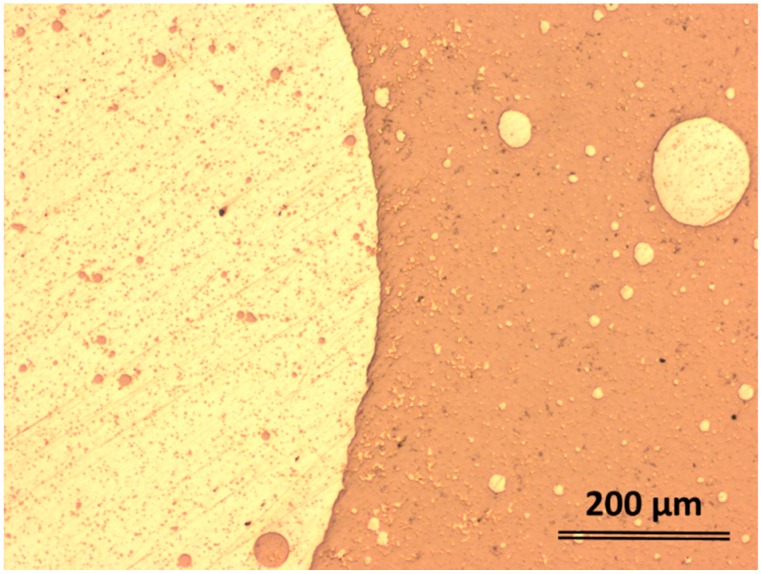
Optical micrographs showing the coarse separation from the liquid state. Centrifugal-cast rod with 5 mm diameter.

Altogether, it is therefore not wrong to suppose that the entire alloy may be treated as a pseudo-binary (Ag,Sn,Pd)-(Ti,Zr,Pd) alloy. The casting features are completed by the further experimental investigations. Figure 2 presents an optical micrograph (OM) of the coarse separation in the 5 mm diameter rod. Even if the alloy was quenched from the homogeneous liquid state, as it is in this actual case, the cooling rate is not high enough to freeze that state and obtain a fully amorphous phase. The spherical appearance and the morphology indicate that the precipitation took place in the liquid state and then it was frozen upon quenching. Then, each domain developed its own microstructure, as it will be shown later. The 5 mm diameter rods are characterized by a hard outer shell, the dark contrast in Figure 2, and a soft core, as illustrated by the light contrast in Figure 2. Judging from the appearance, it is reasonable to suppose that the soft core is very rich in Ag, while the hard margins are Ti rich. Hence the coexistence of liquid and solid phases can be assumed to have the root in the Ag-Ti phase diagram, but nevertheless the new quinary Ti_42_Zr_10_Pd_14_Ag_26_Sn_8_ liquid alloy has multiple atomic interactions.

### 2.2. Structural Characterization

**Figure 3:**
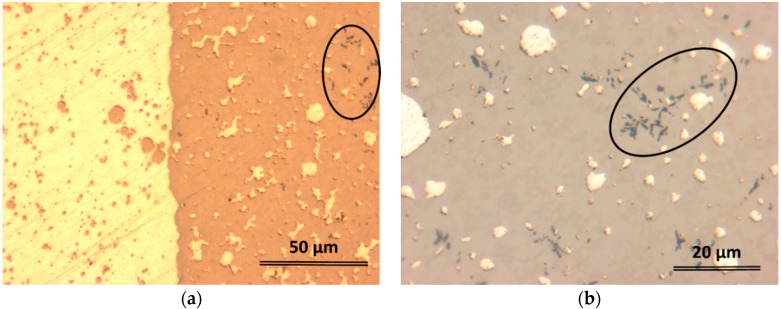
Optical micrographs showing the phase separation in (**a**) 5 mm diameter rod; and (**b**) 2 mm diameter rod.

**Figure 4:**
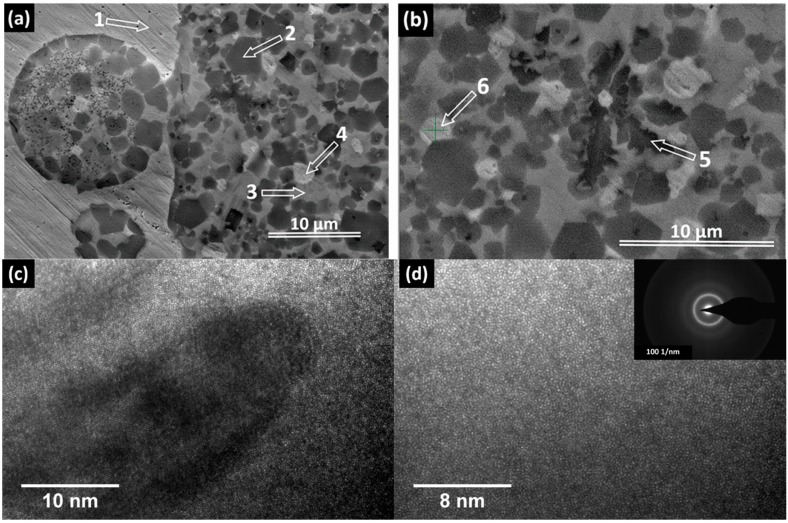
SEM and HR-TEM micrographs and position of characteristic zone for EDX analysis. (**a**) Transversal section trough 5 mm diameter rod, SEM; (**b**) Transversal section trough 2 mm diameter rod, SEM; (**c**) HR-TEM micrograph showing the interface between the amorphous zone (3) and an adjacent crystalline area; (**d**) HR-TEM micrographs, showing in detail only the amorphous area. The inset represent the corresponding selected area diffraction pattern (SAED).

As mentioned previously, the 5 mm diameter rods are characterized by a soft inner core and a much harder outer shell. Figure 3a shows the appearance of the border zone between such two areas (optical microscopy, transversal section). The brighter area is the soft zone, while the darker area is the hard zone. Both dark and bright phases also embed globules that seem to be secondary separations of the same constituents. Additionally, one can observe the formation of some dendrites (marked by an ellipse in both Figure 3a,b). Unlike the 5 mm sample, the smaller rods with 2 and 3 mm diameter did not form large distinct bright and dark areas, only separations between the bright-colored phase inside the dark matrix, similarly to 5 mm rod (see Figure 3b). The higher cooling rate attained during casting by the rods with smaller diameters made the precipitated features to be finer, thus for the clarity, the OM image in Figure 3b was taken at higher magnification (see the scale bar). The larger magnification reveals not only the existence of another phase, the black-colored dendritic-shape as marked in both Figure 3a,b, but also the presence of a much finer structure embedded in the dark phase. Scanning electron microscopy (SEM) using back-scattered electrons (BSE) mode, and energy dispersive X-ray analysis (EDX) were performed on samples extracted from transversal section 5 mm and 2 mm diameter rods, using a FEI Quanta 250 FEG scanning electronic microscope (FEI, Eindhoven, The Netherlands) equipped with an EDAX SDD Apollo X sensor (EDAX Inc., Mahwah, NJ, USA). Considering the complexity of resulted microstructure, quantitative investigations were performed separately on characteristic zones in order to determine compositions of microstructural constituents. Large scale magnifications by mean of SEM (Figure 4) reveal the complex nature of dark constituent evidenced by mean of optical microscopy, as well as homogeneous appearance of the bright phase.

Several features can be easily identified in Figure 4. The bright, mechanically soft area (see the polishing scratches) labelled there with 1, should be the bright area as seen in the optical micrographs. The EDX analyses confirmed that it is almost 100% Ag, as it was supposed previously. The harder zone contains polygonal crystals with hexagonal appearance (labelled with 2), which may be an intermetallic phase. There are also other brighter phases, labelled with 4 and 6, then a darker dendrite (labelled with 5) and the matrix (labelled with 3). Finer dendrites are observed also in the spherical separation (left side of Figure 4a), but they were not marked here in order to keep the micrographs as clear as possible. The EDX analysis of all these features revealed the corresponding elemental composition and the data are summarized in Table 1.

As concluded in Section 2.1 and based on thermodynamic aspects, the early precipitation is indeed Ag-rich in a TiZrPdSn liquid matrix. The bright zones 4 and 6 are again Ag rich and they are formed most probably by a secondary precipitation during rapid quenching. The dendrite (*i.e.*, the dark fifth zone) is almost 50-50 Ti-Zr, therefore it is not wrong to suppose that it is a bcc β-Ti type solid solution. The intermetallic phase, marked in Figure 4 with 2, has a more complex composition. Nevertheless, judging from all possible combinations, the most plausible one is of the type Ti_3.2_Pd_0.8_. The featureless gray area marked here with 3 seems to have the approximated composition Ti_29.26_Zr_32.72_Pd_24.14_Ag_7_Sn_6.88_. Judging from the absence of any features, it is to suppose that this matrix is amorphous. Nevertheless, the SEM magnification is not high enough to rule-out the presence of additional small crystalline features. Further studies, as high-resolution transmission electron microscopy (HR-TEM) were performed. Figure 4c shows in details the interface between matrix and one of the crystalline feature present around. As it can be observed, the matrix appears to be featureless. Further micrographs, one of them presented in Figure 4d, clearly proved that the matrix is amorphous. Additional details are given in the inset in Figure 4d, which shows the selected area diffraction pattern (SAED) of the corresponding zone. Additionally, further casting tests with a closed composition, Ti_30_Zr_32_Pd_24_Ag_7_Sn_7_, revealed that fully amorphous ribbons may be fabricated upon melt spinning (results not presented here).

The structure of the rapid-quenched rod samples appears to be relatively heterogeneous, with coarser features as the diameter increases. This is certainly related to the casting parameters and the cooling rates attained by the samples during preparation. The rods with larger diameters are cooled at lower rates as compared to the rods with smaller diameter, therefore the observed microstructural differences (see for example Figure 3). However, as seen in Figure 4, the crystalline precipitations seem to be identical, so the casting parameters influence only the microstructure. As a consequence, further optimization of all parameters during sample preparation may lead toward samples with more homogeneous structure.

### 2.3. XRD Studies and Thermal Characterization

The samples were investigated by X-ray diffraction in Bragg-Brentano configuration (FEI X’Pert PRO MPD diffractometer (PANalytical B.V., Almelo, The Netherlands) with a copper anode X-ray tube having λ = 0.155 nm, PixCEL detector (PANalytical B.V., Almelo, The Netherlands), vertical θ-θ goniometer and spinning sample holder) in continuous scanning mode using a step size of 0.0130 degrees and 80 s step time, at room temperature. The patterns were further analyzed using the X’Pert HighScore Plus software from PANalytical B.V., Almelo, The Netherlands linked with the ICDD PDF-4+ Database provided by International Centre for Diffraction Data, Newtown Square, PA, USA.

**Figure 5:**
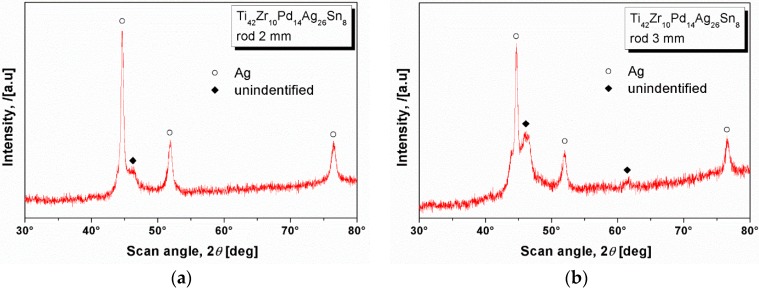
XRD patterns of the Ti_42_Zr_10_Pd_14_Ag_26_Sn_8_ bulk sample with (**a**) 2 mm; and (**b**) 3 mm diameter.

Surprisingly, in contrast with the numerous features as observed in SEM, the XRD revealed only a few features. Figure 5 shows the diffraction pattern stemming from the (a) 2 mm; and (b) 3 mm diameter rod. The main three peaks are characteristic to the fcc-Ag. The patterns show only a few other crystalline peaks, as the one centered at *2*θ = 46° in Figure 5a and *2*θ = 44°, *2*θ = 46° and *2*θ = 62° in Figure 5b. These peaks cannot be identified with acceptable accuracy.

**Figure 6:**
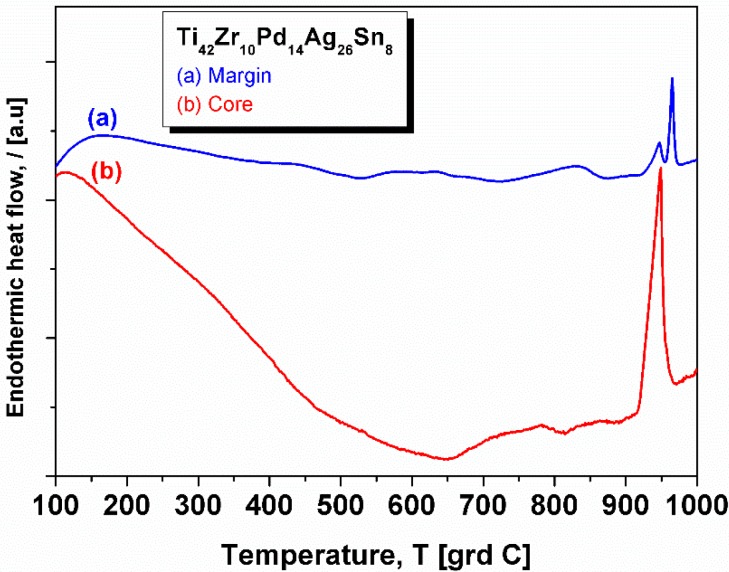
DSC thermograms of the 5 mm diameter rod: (**a**) sample taken from margin; (**b**) sample taken from the core.

Figure 6 presents the thermograms of the 5 mm diameter rod, for both a ductile core and hard margin. There are clear differences, the most notable being huge relaxation up to 650 °C and a single melting peak (curve (b)-the core). Due to the technical limitations, the DSC was performed only up to 1000 °C, the temperature at which, as observed during casting, the alloy is in the liquid + solid state. The liquid temperature of the core (measured as the onset of the melting event) is at 965 °C, proving that this core is almost pure Ag (*i.e.*, melting temperature 962 °C). Therefore, the relaxation-like event may be generated by the annihilation of the mechanical stress induce upon rapid cooling or by the huge thermal expansion that characterizes the Ag metal. The hard margin (curve (a)) shows a double endothermic peak prior 1000 °C. The first of them may be associated with the melting of Ag, stemming from the rest of the Ag trapped in the Ti-rich area and as identified in SEM pictures, while the second one is most probably an allotropic transformation.

**Figure 7:**
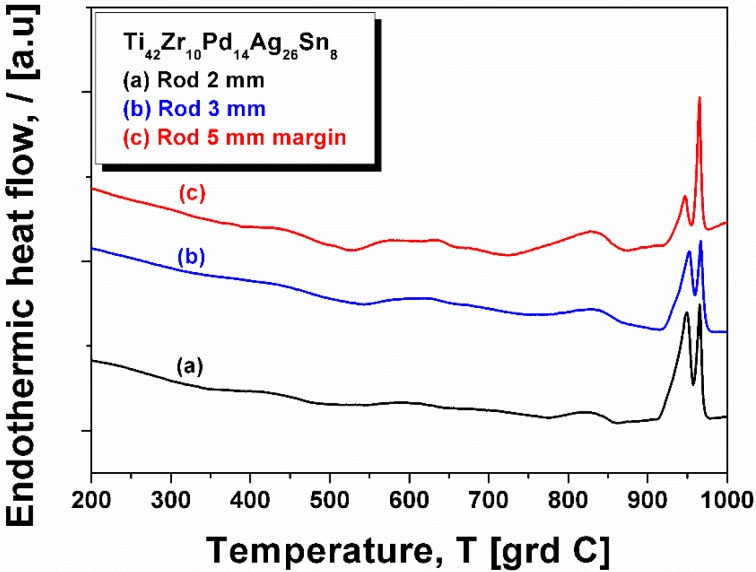
DSC thermograms of rods with 2 mm and 3 mm diameter. For comparison, the thermogram stemming from the margins of 5 mm diameter rod is also presented. (**a**) sample taken from rod with 2 mm diameter; (**b**) sample taken from rod with 3 mm diameter; (**c**) sample taken from a margin of a 5 mm diameter rod.

The curve also shows a small exothermic event at around 550 °C (which may be associated with the crystallization of the remnant amorphous phase) and a small endothermic peak centered at approximatively 840 °C. This peak indicate an allotropic transformation as well, and, together with the exothermic one, it is also present in the thermograms measured for 2 mm and 3 mm diameter rods (Figure 7, curves (a) and (b), respectively). For better comparison, the DSC curve of the hard margin of the 5 mm diameter rod is re-plotted in Figure 7 (curve (c)). All curves show the melting event characteristic of Ag, more pronounced in the case of 2 mm and 3 mm diameter rods, because there the precipitated Ag is better dispersed.

## 3. Materials and Methods

Cylindrical rods with 2, 3, and 5 mm diameter and atomic composition Ti_42_Zr_10_Pd_14_Ag_26_Sn_8_ were produced by mean of two-step fabrication technique. Preliminarily, several ingots weighing maximum 10 g of master alloy were prepared using high purity elements (99.9% and better) by arc melting under Ti-gettered 99.998% argon atmosphere. Each ingot was flipped and re-melted several times to assure the mixing. The master alloy ingots presented heterogeneous microstructure, characterized by ductile outer skin and fragile cores, indicating the separation of Ag. Therefore, in order to minimize errors due to the master alloy heterogeneity, each ingot was used for one single casting trial. The ingots were re-melted in a graphite crucible by induction and then centrifugally cast into a copper mold. In order to avoid the separation, the casting was performed from a temperature of 1520 °C, where the liquid looked homogeneously. The samples were investigated by X-ray diffraction in Bragg-Brentano configuration, using an FEI X’Pert PRO MPD diffractometer (PANalytical B.V., Almelo, The Netherlands) with a copper anode X-ray tube having λ = 0.155 nm, equipped with a PixCEL detector (PANalytical B.V., Almelo, The Netherlands), vertical θ-θ goniometer and spinning sample holder, in continuous scanning mode using a step size of 0.0130 degrees and 80 s step time, at room temperature. The patterns were further analyzed using the X'Pert HighScore Plus software (PANalytical B.V., Almelo, The Netherlands) linked with the ICDD PDF-4+ Database (International Centre for Diffraction Data, Newtown Square, PA, USA). The thermal behavior of the cast samples was determined by heating small samples of the material up to a temperature of 1000 °C under Ar flow in a Netzsch differential scanning calorimeter (DSC). The heating rate was set to 20 K/min. The temperature was limited due to technical reasons: in a liquid state, Ag evaporates and further deposits onto the measuring head. Due to its very high electrical conductivity, it makes bridges between different electrical wirings and makes the continuation of the measurement impossible. Scanning electron microscopy (SEM) using back-scattered electrons (BSE) mode, and energy dispersive X-ray analysis (EDX) were performed on samples extracted from transversal section 5 mm and 2 mm diameter rods, using a FEI Quanta 250 FEG scanning electronic microscope (FEI, Eindhoven, The Netherlands) equipped with an EDAX SDD Apollo X sensor (EDAX Inc., Mahwah, NJ, USA). The high-resolution transmission electron microscopy (HR-TEM) investigations were carried out at 200 kV accelerating voltage on a FEI Tecnai G2 200 kV S/TEM microscope (FEI, Eindhoven, The Netherlands). The TEM samples were prepared with a FEI Quanta 3D dual beam microscope (FEI, Eindhoven, The Netherlands), using focused beams of gallium ions.

## 4. Conclusions

The alloy design by means of molecular orbital method seems to have limitations, and replacement of Cu with Ag did not result in a fully amorphous structure. The resulting Ti_42_Zr_10_Pd_14_Ag_26_Sn_8_ alloy samples prepared by means of copper mold centrifugal casting method reveal a composite structure, where only remainders of amorphous matrix are present. More interestingly, crystalline fraction is represented predominantly by metallic Ag, which separates as small globules with dimensions within few micrometers of range. This microstructural configuration could take full advantage of bactericide effect of metallic silver.

## Figures and Tables

**Table 1:** Atomic compositions of labelled zones in Figure 4.

Element	Zone 2	Zone 3	Zone 4	Zone 5	Zone 6
Pd	13.60	24.14	0.96	10.89	4.32
Ag	3.97	7.00	75.06	2.28	53.51
Sn	7.54	6.88	1.66	6.37	3.60
Ti	52.37	29.26	0.85	39.93	14.34
Zr	22.51	32.72	21.47	40.54	24.23
Total	100	100	100	100	100

## Abbreviations

The following abbreviations are used in this manuscript:

DSC	differential scanning calorimetry
XRD	X-ray diffraction
OM	optical microscopy
SEM	scanning electron microscopy
HR-TEM	high-resolution transmission electron microscopy
SAED	selected area diffraction pattern
BSE	back-scattering electrons
EDX/EDAX	energy-dispersive X-ray analysis
ICDD PDF4+	International Center for Diffraction Data Powder Diffraction File

## Appendix A. Theoretical Design of New Alloy Composition

Composition of new biocompatible alloys is determined using the molecular orbital method for the bcc Ti, based on two alloying parameters, bond order (*B_o_*) and the metal d-orbital energy level (*M_d_*), which are determined theoretically. Parameter *B_o_* measures the covalent bond strength between Ti and an alloying element, while *M_d_* correlates the electronegativity and the metallic radius of elements.

The values $\bar{B_{o}}$ and $\bar{M_{d}}$ for the alloys are determined as atomic compositional averages of the parameters for each pure metal used as alloying element, calculated by Morinaga [32]:

(A1) $$\bar{B_{o}}=\overset{n}{\underset{i=1}{\sum }}x_{i}{\left(B_{o}\right)}_{i}$$

(A2) $$\bar{M_{d}}=\overset{n}{\underset{i=1}{\sum }}x_{i}{\left(M_{d}\right)}_{i}$$

Coordinates $\bar{B_{o}}$ and $\bar{M_{d}}$ are locating each alloy inside a distribution chart area corresponding to an estimated structural state: amorphous, glassy, BMG, crystalline *etc.* [33]. The chart portion belonging to the area of bulk amorphous glasses is shown in Figure A1 and the list of $\bar{B_{o}}$ and $\bar{M_{d}}$ for some alloying elements are given in Table A1.

The reference alloy for development of new copper-free compositions is the BMG with atomic composition Ti_45_Zr_10_Pd_10_Cu_31_Sn_4_, having a critical diameter of 4 mm and compression strength of 1970 MPa [29]. The parameters determined by the molecular orbital method are $\bar{B_{o}}$ = 2.532 and $\bar{M_{d}}$ = 1.689, which situate the alloy inside the BMG area (see the position of the round dot in Figure A1), confirming the d-electron alloy design theory. Replacement of copper with silver requires that the resulting alloy remain inside the BMG area, therefore final composition was optimized to produce appropriate values of $\bar{B_{o}}$ and $\bar{M_{d}}$. Optimization results are presented in Table A2, while in Figure A1 the square symbol corresponding to the new alloy is positioned inside BMG domain.

**Table A1:** *B*_o_ and *M_d_* values for alloying elements in bcc Ti [32].

Element	*B_o_*	*M_d_*** (eV)
Ti	2.790	2.447
Zr	3.086	2.934
Pd	2.208	0.387
Cu	2.114	0.567
Ag	2.094	0.196
Sn	2.283	2.100
Nb	3.099	2.424

**Table A2:** Compositions and orbital method parameters of considered alloys.

Alloy	Ti (% at.)	Zr (% at.)	Pd (% at.)	Cu (% at.)	Ag (% at.)	Sn (% at.)	Bo	Md (eV)
Ti_45_Zr_10_Pd_10_Cu_31_Sn_4_	45	10	10	31	–	4	2.532	1.689
Ti_42_Zr_10_Pd_14_Ag_26_Sn_8_	42	10	14	–	26	8	2.517	1.589

## Appendix B. Biological Effect of Copper and Silver

Biocompatibility evaluation of different materials, which are in contact with the human body for long periods, is a very complex issue [3]. Allergenic, cytotoxic, and inflammatory effects are some of the most important properties that are usually investigated for assessment of metallic alloys for biomedical use [1,2]. Unlike nickel, cobalt, or chrome, neither copper nor silver have been reported so far for significant allergenic potency [37]. Therefore, studies are focused on the cyto-compatibility and possible inflammatory reactions. Material cytotoxicity is most commonly estimated *in vitro* by relative viability, proliferation, and morphological changes of different cell lines in contact with metallic alloys for some periods of time, while the test of inflammatory reactions consisted of quantifying the multinucleated giant cells (MGC) appearing in monolayer cell cultures. These results could be aggregated with measurements of the rates on which ions of alloying elements are released from biomaterials, since metallic ions released by biomedical implants represent the main cause of unwanted secondary reactions in organisms. The resulting cytotoxicology index (CI) allows scoring of metallic alloys between CI = 0, when the tested alloy is highly cytocompatible, and CI = 100 for highly cytotoxic or even lethal materials or substances. Although effects of pure copper and silver may be comparable in terms of *in vitro* viability of cell cultures, the ranking by the CI reveals the most unfavorable effect of copper-containing alloys, while the presence of silver as an alloying element is not an unfavorable factor, mostly because the silver ion release rate seems to be very low. For example, a palladium-based alloy containing 28 mass percent of silver has a CI of only 18, while a palladium alloy with 20 mass percent of copper has a CI of 43 [38]. Cytotoxicity ranking of casting alloys evaluated by cell culture tests revealed that Au, Pd, and Ti were the least cytotoxic (rank 1), followed by Ag (rank 2), then Ni (ranked 4), and finally Cu (rank 5), which is the most cytotoxic [39]. *In vitro* experiments of long-term and low-dose exposure to metallic ions determined that Ag^+^ and Hg^2+^ did not produce any deterioration of cell cultures even after four weeks, while the increase of cell death for Cu^2+^ and Ni^2+^ was significant [40].

Major concerns about biosafety of silver lead recently to more detailed studies, which concluded that silver neither had cytotoxicity nor resulted in inflammatory expression if effective concentration of released ions into the organism is maintained within certain limits, specific for each type of organism [41]. Only a few relatively alarming results have been reported so far, exclusively on dental alloys with high silver content, indicating that silver may have, in the worst conditions, only intermediate cytotoxic effect, while copper is considered a highly toxic element, belonging to the same category as zinc and nickel [42]. A more general classification by tissue reaction around metallic implants considers reaction to silver of “capsule” type, triggering only moderate reactions from organisms, in same category as metals like aluminum, iron, molybdenum, and gold, but also consecrated biomaterials such as stainless steels or Co-Cr alloys [43]. Meanwhile, copper belongs to the “toxic” group together with vanadium, nickel, and cobalt. Members of the highly biocompatible “vital” response category are platinum, tantalum, niobium, titanium, and zirconium [44]. Most worrying, some reports evidence genotoxicity of copper, which could be responsible for DNA damage, via some oxidative mechanisms, similar as cadmium, chromium, mercury, nickel, vanadium, and lead [45].

The most recent reference works on metallic implant biomaterials consider that, although health effects of silver are still disputed, metallic silver is non-toxic, while only some silver salts are. Meanwhile, apart from cytotoxic effect, excessive copper amounts have been linked to neurodegenerative diseases (Alzheimer, Menkes, Wilson) [2].

At the same time, germicidal and antimicrobial effects of silver ions are frequently emphasized. It has been documented that silver not only reduces bacterial adhesion, but also inhibits bacterial growth, having pregnant bacteriostatic effect on highly dangerous bacteria such as *Bacillus subtilis*, *Escherichia coli, etc.* [41,46]. Anti-biofilm activity was reported against *Pseudomonas aeruginosa* and *Staphylococcus epidermidis* [47]. Silver ions were determined to be non-toxic, safe antibacterial for human body, within certain limits. They also possess anti-fungal activity, anti-inflammatory effects, and anti-viral and anti-angiogenic activity. It may be added that almost all research stresses that safely applied therapies imply control on released concentrations of silver ions [47].

It may be concluded that, considering necessary precaution about resulting concentrations of released ions, silver has already won considerable attention for applications for use in cosmetics, ophthalmology, alloying elements for fabrication of anti-microbial surgical tools, additives for synthetic elastomers in medicine, and even coatings on titanium implants [48]. Therefore, it may be considered that using silver as amorphization element for fabrications of titanium-based BMGs may be reasonable from the point of view of health safety, with respect of some precautions and as a result of specific investigations regarding *in vivo* biocompatibility for certain applications.

## References

[1] Abdel-Hady M.G., Niinomi M. (2013). Biocompatibility of Ti-alloys for long-term implantation. J. Mech. Behav. Biomed. Mat..

[2] Chen Q., Thouas G.A. (2015). Metallic implant biomaterials. Mat. Sci. Eng. R.

[3] Niinomi M. (2010). Metals for Biomedical Devices.

[4] Niinomi M. (2008). Mechanical biocompatibilities of titanium alloys for biomedical applications. J. Mech. Behav. Biomed. Mat..

[5] Biesiekierski A., Wang J., Gepreel M.A.-H., Wena C. (2012). A new look at biomedical Ti-based shape memory alloys. Acta. Biomat..

[6] Calin M., Gebert A., Ghinea A.C., Gostin P.F., Abdi S., Mickel C., Eckert J. (2013). Designing biocompatible Ti-based metallic glasses for implant applications. Mat. Sci. Eng. C.

[7] Niinomi M. (1999). Recent Titanium R&D for Biomedical Applications in Japan. J. Min. Met. Mat. Soc..

[8] Greer A.L. (2009). Metallic glasses... on the threshold. Mat. Today.

[9] Axinte E. (2012). Metallic glasses from “alchemy” to pure science: Present and future of design, processing and applications of glassy metals. Mat. Des..

[10] Wang W., Dong C., Shek C. (2004). Bulk metallic glasses. Mat. Sci. Eng. R.

[11] Eckert J., Das J., Pauly S., Duhamel C. (2007). Mechanical properties of bulk metallic glasses and composites. J. Mater. Res..

[12] Schroers J., Kumar G., Hodges T.M., Chan S., Kyriakides T.R. (2009). Bulk Metallic Glasses for Biomedical Applications. JOM.

[13] Nicoara M., Raduta A., Parthiban R., Locovei C., Eckert J., Stoica M. (2016). Low Young’s modulus Ti-based porous bulk glassy alloy without cytotoxic elements. Acta. Biomat..

[14] Klement W., Willens R.H., Duwez P. (1960). Non-crystalline structure in solidifed gold-silicon alloys. Nature.

[15] Inoue A. (2000). Stabilization of metallic supercooled liquid. Acta. Mater..

[16] Peker A., Johnson W. (1994). Beryllium Bearing Amorphous Metallic Alloys Formed. US Patent.

[17] Inoue A., Takeuchi A. (2011). Recent development and application products of bulk glassy alloys. Acta. Mat..

[18] Zhang T., Inoue A. (2001). Ti-based amorphous alloys with a large supercooled liquid region. Mat. Sci. Eng..

[19] Zhang T., Inoue A. (1998). Thermal and Mechanical Properties of Ti-Ni-Cu-Sn Amorphous Alloys with a Wide Supercooled Region before Crystallization. Mat. Trans. JIM.

[20] Guo F., Wang J., Poon J.S., Shiflet G.J. (2005). Ductile titanium-based glassy alloy ingots. Appl. Phys. Lett..

[21] Tang M.Q., Zhang H.F., Zhu Z.W., Fu H.M., Wang A.M., Li H., Hu Z.Q. (2010). TiZr-base Bulk Metallic Glass with over 50 mm in Diameter. J. Mater. Sci. Technol..

[22] Zhu S.L., Wang X.M., Qin F.X., Yoshimura M., Inoue A. (2007). New TiZrCuPd Quaternary Bulk Glassy Alloys with Potential of Biomedical Applications. Mat. Trans..

[23] Wang H., Park E.S., Oak J.J., Setyawan A.D., Zhu S.L., Wada T., Wang X.M., Takeuchi A., Kato H. (2013). Effect of cobalt microalloying on the glass forming ability of Ti-Cu-Pd-Zr metallic-glass. J. Non Cryst. Sol..

[24] Suo Z., Qiu K., Li Q., Ren Y., Hu Z. (2010). Effect of Nb on glass forming ability and plasticity of (Ti-Cu)-based bulk. Mat. Sci. Eng. A.

[25] Zhu S., Wang X., Inoue A. (2008). Glass-forming ability and mechanical properties of Ti-based bulk. Intermetallics.

[26] Oak J.-J., Louzguine-Luzgin D.V., Inoue A. (2007). Fabrication of Ni-free Ti-based bulk-metallic glassy alloy having potential for application as biomaterial, and investigation of its mechanical properties, corrosion, and crystallization behavior. J. Mat. Res..

[27] Zheng N., Qu R.T., Pauly S., Calin M., Gemming T., Zhang Z.F., Eckert J. (2012). Design of ductile bulk metallic glasses by adding “soft” atoms. Appl. Phys. Lett..

[28] Qin F.X., Wang X.M., Inoue A. (2007). Effects of Ta on Microstructure and Mechanical Property of Ti-Zr-Cu-Pd-Ta Alloys. Mat. Trans..

[29] Oak J.-J., Kimura H., Inoue A. (2007). Effects of Additional Elements on Structure, Mechanical Strength and Chemical Properties of Ni-free Ti-based Bulk Metallic Glasses for Biomaterials. Adv. Mat. Res..

[30] Qin F., Wang X., Xie G., Inoue A. (2008). Distinct plastic strain of Ni-free Ti-Zr-Cu-Pd-Nb bulk metallic glasses with potential for biomedical applications. Intermetallics.

[31] Oak J.-J., Hwang G.-W., Park Y.-H., Kimura H., Yoon S.-Y., Inoue A. (2009). Characterization of Surface Properties, Osteobalst Cell Culture *in Vitro* and Processing with Flow-Viscosity of Ni-Free Ti-Based Bulk Metallic Glass for Biomaterials. J. Biomech. Sci. Eng..

[32] Abdel-Hady M.M., Hinoshita K., Morinaga M. (2006). General approach to phase stability and elastic properties of beta-type Ti-alloys using electronic parameters. Scr. Mat..

[33] Oak J.-J., Louzguine-Luzgin D.V., Inoue A. (2007). Synthetic relationship between titanium and alloying elements in designing Ni-free Ti-based bulk metallic glass alloys. Appl. Phys. Lett..

[34] Goodman S.B., Yao Z., Keeney M., Yang F. (2013). The future of biologic coatings for orthopaedic implants. Biomaterials.

[35] Takeuchi A., Inoue A. (2005). Classification of bulk metallic glasses by atomic size difference, heat of mixing and period of constituent elements and its application to characterization of the main alloying element. Mat. Trans..

[36] Villars P., Okamoto H., Cenzual K. ASM Alloy Phase Diagrams Database. http://www.asminternational.org.

[37] Schlede E., Aberer W., Fuchs T., Gerner I., Lessmann H., Maurer T., Rossbacher R., Stropp G., Wagner E., Kayser D. (2003). Chemical substances and contact allergy—244 substances ranked according to allergenic potency. Toxicology.

[38] Hornez J., Lefevre A., Joly D., Hildebrand H. (2002). Multiple parameter cytotoxicity index on dental alloys and pure metals. Biomol. Eng..

[39] Craig R., Hanks C. (1990). Cytotoxicity of Experimental Casting Alloys Evaluated by Cell Culture Tests. J. Dent. Res..

[40] Wataha J., Lockwood P., Schedle A. (2000). Effect of silver, copper, mercury, and nickel ions on cellular proliferation during extended, low-dose exposures. J. Biomed. Mat. Res..

[41] Hsu S.-H., Tseng H.-J., Lin Y.-C. (2010). The biocompatibility and antibacterial properties of waterborne polyurethane-silver nanocomposites. Biomaterials.

[42] Elshahawy W.M., Watanabe I., Kramer P. (2009). *In vitro* cytotoxicity evaluation of elemental ions released from different prosthodontic materials. Dent. Mat..

[43] Long M., Rack H. (1998). Titanium alloys in total joint replacement—A materials science perspective. Biomaterials.

[44] Kuroda D., Niinomi M., Morinaga M., Kato Y., Yashiro T. (1998). Design and mechanical properties of new β type titanium alloys for implant materials. Mat. Sci. Eng. A.

[45] Stohs S., Bagchi D. (1995). Oxidative mechanisms in the toxicity of metal ions. Free Radic. Biol. Med..

[46] Choi O., Yu C.-P., Fernandez G.E., Hua Z. (2010). Interactions of nanosilver with *Escherichia coli* cells in planktonic and biofilm cultures. Water Res..

[47] Kalishwaralal K., BarathManiKanth S., Pandian S.R.K., Deepak V., Gurunathan S. (2010). Silver nanoparticles impede the biofilm formation by *Pseudomonas aeruginosa* and *Staphylococcus epidermidis*. Colloids Surf. B.

[48] Secinti K.D., Özalp H., Attar A., Sargon M.F. (2011). Nanoparticle silver ion coatings inhibit biofilm formation on titanium implants. J. Clin. Neurosci..

